# Wound-Healing Effects of Curcumin and Its Nanoformulations: A Comprehensive Review

**DOI:** 10.3390/pharmaceutics14112288

**Published:** 2022-10-25

**Authors:** Amrita Kumari, Neha Raina, Abhishek Wahi, Khang Wen Goh, Pratibha Sharma, Riya Nagpal, Atul Jain, Long Chiau Ming, Madhu Gupta

**Affiliations:** 1 School of Pharmaceutical Sciences, Delhi Pharmaceutical Sciences and Research University (DPSRU), New Delhi 110017, India; 2 Faculty of Data Science and Information Technology, INTI International University, Nilai 71800, Malaysia; 3 University Institute of Pharmaceutical Sciences, Panjab University, Chandigarh 160014, India; 4 Department of Pharmaceutics, Delhi Institute of Pharmaceutical Sciences and Research, Delhi Pharmaceutical Sciences and Research University (DPSRU), New Delhi 110017, India; 5 PAPRSB Institute of Health Sciences, Universiti Brunei Darussalam, Gadong BE1410, Brunei

**Keywords:** drug delivery, antioxidant, skin, wound healing, nanotechnology, antibiofilm, polyphenolic compound, FDA regulations, Patents, Diabetic foot ulcer

## Abstract

Wound healing is an intricate process of tissue repair or remodeling that occurs in response to injury. Plants and plant-derived bioactive constituents are well explored in the treatment of various types of wounds. Curcumin is a natural polyphenolic substance that has been used since ancient times in Ayurveda for its healing properties, as it reduces inflammation and acts on several healing stages. Several research studies for curcumin delivery at the wound site reported the effectiveness of curcumin in eradicating reactive oxygen species and its ability to enhance the deposition of collagen, granulation tissue formation, and finally, expedite wound contraction. Curcumin has been widely investigated for its wound healing potential but its lower solubility and rapid metabolism, in addition to its shorter plasma half-life, have limited its applications in wound healing. As nanotechnology has proven to be an effective technique to accelerate wound healing by stimulating appropriate mobility through various healing phases, curcumin-loaded nanocarriers are used for targeted delivery at the wound sites. This review highlights the potential of curcumin and its nanoformulations, such as liposomes, nanoparticles, and nano-emulsions, etc. in wound healing. This paper emphasizes the numerous biomedical applications of curcumin which collectively prepare a base for its antibiofilm and wound-healing action.

## 1. Introduction

The skin is the largest organ of the human body, comprising 15% of a person’s body weight and with a surface area of about 20 square feet [1]. It protects the internal structures from any kind of physical, biological, chemical, and mechanical stress from the outer environment. It is also involved in thermoregulation, immune-regulatory observation, water loss prevention, sensation, and vitamin cholecalciferol (D_3_) agglutination [2,3,4]. Any damage or disorder in the healthy structure of skin is described as a wound. Wounds can be classified according to their location, depth, etiology, injury type, and appearance [5]. Clinically wounds are classified as chronic or acute wounds. Acute wounds spontaneously heal in about 8–12 weeks, whereas chronic wounds require a longer healing time (sometime even some months) due to prolonged inflammation. Age, obesity, injuries, and chronic ailments, such as diabetes, cancer, and other factors, contribute to chronic wounds [6,7]. Wound healing is an intricate physiological method consisting of coordinated overlapping phases of hemostasis, inflammation, proliferation, and tissue remodeling [8]. Hemostasis is the first and foremost reaction within the first few minutes of the injury. At the injured site, the inflammatory cells and platelets begin to adhere and activate fibrin, forming a mesh-like structure that acts as “glue” to bind platelets with each other. This aggregate develops a clot and along with vasoconstriction helps to prevent further bleeding [9]. The second phase of wound healing is the inflammation that occurs concomitantly with the hemostasis phase. Activation of the complement cascade occurs when a fibrin clot forms, allowing neutrophils to move to the wound site to scavenge bacteria and prepare for healing. The phagocytic cell secretes transforming growth factor β (TGF-β), which initiates a vital signal for the onset of healing at the wound site. The blood monocytes and lymphocytes turn into tissue macrophages, leading to the release of growth factors and cytokines named fibroblast growth factor (FGF), tumor necrosis factor-alpha (TNF-α), and interleukin-1 (IL-1) and, together, these attract endothelial cells, keratinocytes, and fibroblasts to rebuild the damaged blood vessels. Proliferation is the third phase, and during this phase, the contraction of the wound and the replacement of subdermal and dermal tissues take place. Angiogenesis, epithelialization, and collagen production are all initiated by fibroblast cells. TGF-β platelets and macrophages induce collagen production in fibroblasts. In this process, the final phase is remodeling in the healing process; it comprises of new layer formation by fibroblast cells of skin on the wound surface, and the original collagen type III in the injured site is replaced by collagen type I to be cross-linked and realigned along the tension lines [9,10]. There are various genetic factors and acquired factors, such as diabetes, which disrupt the wound healing process. The wounds in diabetic patients are characterized by increased inflammation and abnormal cellular infiltration, faulty cytokine production, neuritis, and inadequate neo-angiogenesis. Increasing health care costs, an aging population, biofilm formation, and the continued threat of diabetes and obesity worldwide make chronic wounds a substantial clinical, social, and economic challenge. According to the 2018 retrospective analysis, Medicare beneficiaries identified that approximately 8.2 million people are suffering from wounds. The annual wound care products market is estimated to reach $15–22 billion by 2024. In the United States alone, around 5.7 million patients are suffering from chronic wounds with an annual cost of treatment of about USD 20 billion [11]. This all leads to the conclusion that there is a huge scope and requirement for research into wound healing. Plants have always been an integrated and most widely studied area in this regard as they promote natural repair mechanisms [12]. With the advancement in science, interest has shifted from whole plants to active chemical constituents. Curcumin is at the top in the wound healing area. Curcumin is a naturally occurring low-molecular-weight polyphenolic constituent present in the rhizome of *Curcuma longa* and *Curcuma aromatic* [13]. Curcumin (77%) is the most prominent bioactive constituent of turmeric rhizomes, followed by demethoxycurcumin (17%), bisdemethoxycurcumin (3%), and cyclocurcumin (3%). It has been used for ages as traditional medicine in the treatment of inflammation and healing of impaired wounds. The topical application of curcumin is documented to have an effective role in wound healing mechanisms. Curcumin acts in different stages, such as the inflammatory, maturation and proliferative phases and thus enhances the overall process of wound healing. However, some limiting factors, such as poor bioavailability, low solubility in water, and rapid metabolism hinder curcumin’s therapeutic efficacy. Toxicity at high concentrations in its topical application is another disadvantage of curcumin [13,14,15]. Hence incorporation of different nano delivery systems to modulate the limiting factors of curcumin is an interesting area to explore to unlock all the possibilities related to this compound in the area of wound healing. The present review highlights the role and importance of curcumin and its nanoformulations for wound healing. Various pharmacological activities associated with it are also covered in the text.

## 2. Chemistry and Applications of Curcumin

**Figure 1:**
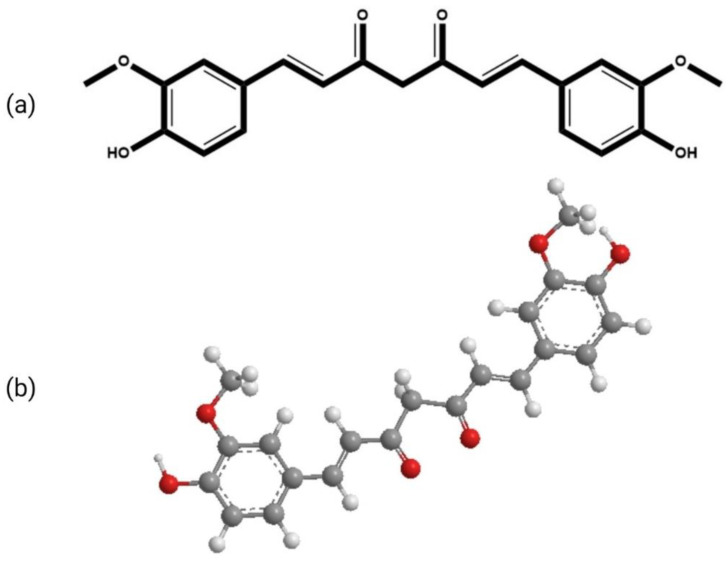
The chemical structure of curcumin (**a**) 2-D (**b**) 3-D.

The molecular formula of curcumin is C_21_H_20_O_6_. Its structure is composed of three chemical entities as shown in Figure 1: two aromatic rings with methoxyl and hydroxyl groups at the ortho position linked by a seven-carbon linker that includes an α, β-unsaturated β-diketone moiety [16]. Due to the presence of the diketo group, curcumin exhibits keto-enol tautomerism. The keto form predominates in slightly acidic and neutral conditions [17], whereas the enolic form dominates in alkaline conditions. In solution, it exhibits *cis-trans* isomerism. The *trans*-form is more stable than the *cis*-form due to the placement difference of two phenolic-methoxy groups on the curcumin backbone. The computed dipole moment of curcumin in its ground state is 10.77 D [18]. The logarithmic value of the octanol/water partition coefficient (*log P*) of curcumin is 3.2, thus making it practically insoluble in water but highly soluble in lipids [16,19]. Due to its lipophilic nature, it acquires adequate transmembrane permeability. Curcumin can be incorporated into aqueous solvents with the aid of lipids, surfactants, albumins, and biopolymers, etc. For the incorporation of higher concentrations of curcumin, micelles have shown promising results. However, as surfactants can interfere in biological studies, proper controlled experiments must be performed while using these aqueous solutions in biological systems.

Curcumin is documented to be effective against a variety of chronic diseases, including Alzheimer’s disease, multiple sclerosis, rheumatoid arthritis, atherosclerosis, and others. It has also shown promising results against cataract formation, liver damage, pulmonary toxicity, fibrosis, and impaired wound healing, inhibits thrombosis and suppresses platelet aggregation [20]. It is also established as an antineoplastic, antimicrobial, anti-fungal, anti-carcinogenic, anti-infective, anti-mutagenic, anti-inflammatory, anti-proliferative, anti-aging, anti-amyloid, and anti-hypercholesterolemia agent [21,22].

It inhibits the activation of free radical-activated transcription factor, nuclear factor kappa B (NFκB) [15], cytokine production, and other cellular processes essential for cell survival. It also inhibits the signal transducer and activator of transcription (STAT) proteins. Curcumin-led inhibition of NFκB-DNA binding suppresses the pro-inflammatory molecules matrix metalloprotease 9 (MMP-9) and matrix metalloprotease 3 (MMP-3) and also reduces pro-inflammatory cytokines, such as tumor necrosis factor 1 (TNF-1), interleukin 1 (IL-1), and interleukin 8 (IL-8). Curcumin also binds to the COX-2 (prostaglandin-endoperoxide synthase 2) protein, which reduces COX-2 expression, prostaglandin and thromboxane synthesis [23]. Furthermore, curcumin is a versatile antioxidant molecule that combats free radicals, as it is hypothesized to reduce reactive oxygen species (ROS) production, neutrophil attraction, adhesion, and migration, which leads to the reduction of damage caused by contusion-induced muscle injury [24]. Curcumin is also documented to lower blood cholesterol and sugar levels and improve insulin sensitivity in diabetic people [25,26]. Furthermore, curcumin has also been reported to possess significant antifungal and antibacterial properties against both gram-positive and gram-negative bacteria [27]. Curcumin-encapsulated PEGylated nanoliposomes showed a potential anti-infective therapeutic effect [28]. Besides, it also possesses anti-biofilm and antibacterial properties against *Porphyromonas gingivalis* [29].

## 3. Wound Healing Potential of Curcumin: Mechanism of Healing

**Figure 2:**
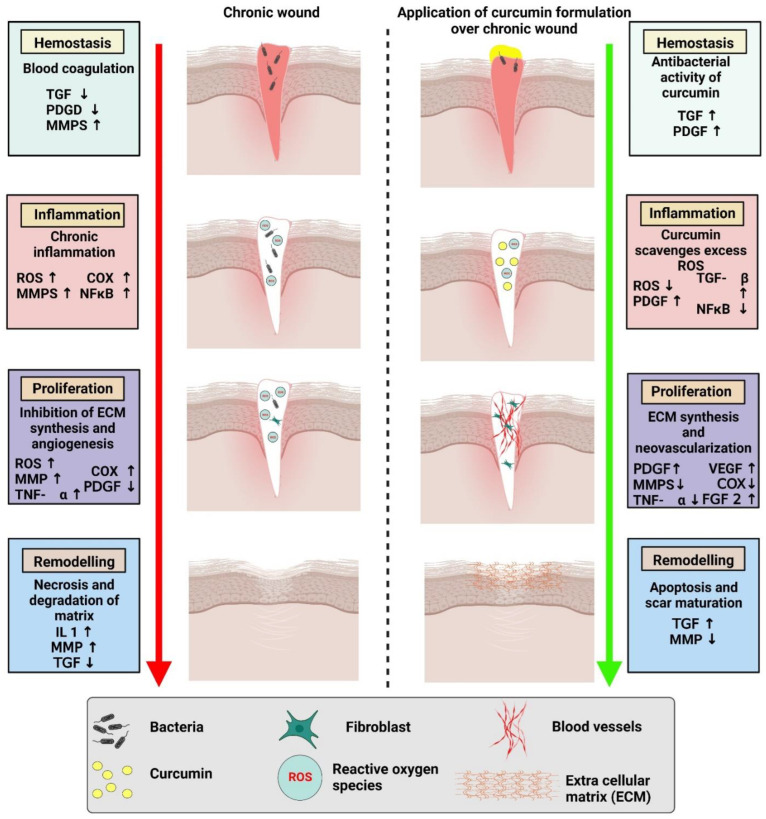
Comparison of chronic wound healing and accelerated wound healing with the application of a curcumin-based formulation.

Of late, biomedical applications give strong evidence for the wound healing potential of curcumin as each and every activity plays an important role in wound healing (Figure 2). Curcumin is a natural polyphenolic antioxidant constituent that has been intensively explored in recent years for its potential as a wound healing agent [30]. The primary objective of wound healing is to restore tissue integrity and maintain homeostasis [31]. Curcumin improves the wound contraction rate, thus accelerating the healing of wounds. It is reported to enhance the wound area significantly up to 20% [32]. Emiroglu et al. have shown that curcumin suppressed the inflammatory response and hastened wound healing [33]. Heydari et al. discovered that curcumin enhanced collagen deposition and promoted angiogenesis in the chronic wound [34].

### 3.1. Effects of Curcumin on Inflammation

Inflammation is the critical second phase of the wound healing process, and it is frequently referred to as the first stage in optimal skin regeneration. Controlling inflammation is desirable and can accelerate the wound healing process since tissue injury produces practically a rapid start of acute inflammation. Curcumin, in particular, has been demonstrated to decrease the production of tumor necrosis factor-alpha (TNF-α) and interleukin-1 (IL-1), two essential cytokines generated by monocytes and macrophages that regulate inflammatory responses. Curcumin’s capacity to block the activity of NF-(κ)B (nuclear factor kappa-light-chain-enhancer of activated B cells), a transcription factor involved in the start of inflammatory reactions, is also significant. Curcumin modulates a variety of pathways associated with the activation of NF-(κ)B, which is generally activated by several kinases (AKT, PI3K, IKK) [35]. For a long time, NF-(κ)B was thought to be oxidant sensitive, emphasizing the link between oxidation and inflammation in wound healing. Other signaling pathways, such as PPAR and myeloid differentiation protein 2-TLR 4 co-receptors, are involved in curcumin’s anti-inflammatory activities (TLR4-MD2) [36,37]. Curcumin also reduces angiotensin II-induced inflammatory reactions by inhibiting vascular smooth muscle cell proliferation by increasing PPAR-γ activity. It has also been shown to reduce inflammation by competing with LPS for MD2 binding, effectively inhibiting the TLR4-MD2 signaling complex [38]. ROS are by-products of aerobic respiration that play a role in intracellular signaling, differentiation, apoptosis, cell development, and immunity. ROS have a role in wound healing because they are necessary for the immune system’s defense against microbes. However, chronic exposure to high levels of ROS causes oxidative stress, which can harm human cells severely. In the wound healing process, oxidative stress is a critical component that limits tissue regeneration. It has been discovered that curcumin has an efficient protective function against oxidative stress through modulating lipoxygenases (LPx), mostly by scavenging free radicals. Curcumin’s capacity to transport electrons or easily give H-atoms from two methoxy phenol groups accounts for its antioxidant properties. It also contains a variety of functional groups, such as b-diketone and a large number of p electrons with high electron-shifting properties. Furthermore, curcumin’s ability to attach to metals is thought to be due to its di-ketone structure, and the phenolic hydroxyl (-OH) groups give ROS scavenging capabilities [39]. Furthermore, it boosts the generation and functioning of antioxidants and their components, such as glutathione (GSH). Stimulation of cytoprotective signaling pathways, such as the nuclear factor erythroid 2-related factor 2 (Nrf2) pathway, have been identified as linked molecular mechanisms in wound healing that cause oxidative stress and an increase in ROS species. This Nrf2 regulation mechanism is critical for cell protection against oxidative damage. In response to electrophilic and oxidative stress, the reactive cysteine residues of Keap1 change, resulting in diminished E3 ligase activity, continued Nrf2 accumulation in cells, and significant stimulation of a number of cytoprotective genes [40]. Curcumin works as a Nrf2 activator and thereby decreases oxidative stress by functioning as a direct or indirect antioxidant [41].

### 3.2. Effects of Curcumin on the Proliferative Phase of Wound Healing

Granulation tissue formation and collagen deposition (the production of the extracellular protein matrix), fibroblast proliferation, epithelialization, and apoptosis of undesired cells are all part of the proliferative phase of wound healing [42]. Various studies have examined the impact of curcumin on these processes, as well as the time for wound closure in curcumin-treated animals compared to controls. According to Panchtantram et al., curcumin promoted collagen production and enhanced cellular proliferation at the wound site, as seen by increased DNA, total protein, and type III collagen content in wound tissues [43]. According to a histological investigation by Gopinath et al., curcumin-increased cytokine production causes fibroblasts to migrate to wound sites, resulting in enhanced fibroblast and collagen proliferation [44]. A comparison research employing a skin incision model showed that curcumin supported complete epithelial repair, enhanced angiogenesis during the proliferative phase of wound healing, and faster wound closure [45].

### 3.3. Effects of Curcumin on Fibroblast Proliferation

The migration of fibroblasts into the wound area is necessary for the formation/remodeling of granulation tissue, as well as the creation and deposition of collagen [46]. As a result, fibroblasts in the wound environment are undoubtedly the most significant mediator in guaranteeing quick and aesthetically pleasing wound closure. Fibroblasts naturally develop into myofibroblasts during the creation of granulation tissue. Curcumin treatment has been demonstrated to cause fibroblast infiltration into wound sites in various research findings [47,48]. Ravanfar et al. observed that curcumin dramatically sped up cellular proliferation and shortened the inflammatory phase, showed considerably greater fibroblast distribution/one mm^2^ of wound area, and quick re-epithelialization [48].

### 3.4. Effects of Curcumin on Granulation Tissue Formation

After four days of a skin injury, granulation tissue, or new stroma, starts to form, where small capillaries develop, and fibroblasts infiltrate, facilitating the creation of an extracellular matrix. Curcumin improves granulation tissue formation, which further facilitates re-epithelialization by providing a stable foundation for epithelial cells to migrate and heal the wound gap. A study done by Aslam et al. has shown that granulation tissue was present in the curcumin-treated group, along with mild collagen deposition, neovascularization, and moderate inflammatory cells (D–F). Furthermore, they have also shown that the group that received treatment with curcumin + ZnO nanoconjugates displayed traits that were close to normal, including enlarged collagen bundles in the dermis, severe fibrosis, angiogenesis, and an altered collagen matrix (G–I) [49].

### 3.5. Effect of Curcumin on Collagen Deposition

The extracellular matrix must be reorganized and remodeled in order for wounds to heal entirely. The extracellular matrix, which includes granulation tissue and collagen, gives support to cells and comprises of various proteins and polysaccharides. Collagen is the most abundant protein in the extracellular matrix of the skin, which accounts for 70–80% of its mass. The creation of scar tissue, which is largely made up of collagenous fibers, is the end outcome of wound healing. As a result, sufficient collagen production and deposition in a wound site would be ideal for improving wound repair. Curcumin has been reported to enhance collagen and extracellular matrix synthesis and thereby accelerate the process of wound healing [50,51]. It has been observed that curcumin tends to accelerate wound healing as it can start synthesis and release of collagen on the third day after its application on a wound as shown by Mahmoud et al., 2022 [52]. Qian et al. showed that curcumin can aggregate wound exudate to cause a cascade release of curcumin, which speeds up the healing process while promoting collagen deposition and vascularization [53].

## 4. Action of Curcumin in the Management of Diabetic Foot Ulcers (DFUs)

A severe complication of diabetes known as a diabetic foot ulcer (DFU) is mainly brought on by ischemia, infection, local trauma, diabetic neuropathy, peripheral vascular diseases (PVDs), and diabetic neuropathy [54]. The diabetic ulcer, one of the chronic wounds, has delayed wound healing. In contrast to acute wounds (kept at hemostasis), amputation is the most likely result if DFUs are not treated in the early stages [55]. The DFUs state is made worse by PVDs and decreased blood flow. Today, DFU is treated using a multimodal strategy that includes glucose control, infection eradication, ulcer healing promotion, and high-pressure remission [56,57,58]. It has been shown that in diabetic patients with foot wounds, strict glycemic management dramatically reduces the chance of amputation [59]. Additionally, few research studies on humans and animals have looked at the effects of vitamin and natural component supplements on DFU’s metabolic variables and wound healing [60].

A well-known phyto-constituent called curcumin has been studied for its potential to cure diabetic foot ulcers. Curcumin aids in wound healing by lowering oxidative stress, reducing inflammatory activities, and promoting the proliferation and remodeling stages, as stated in the above section [61]. Further, the activation of PPAR-gamma (PPAR-γ), lipoprotein lipase (LPL), and low-density lipoprotein receptor (LDLR) may increase insulin sensitivity, glucose absorption in muscle and adipose tissue and improve lipid homeostasis, contributing to curcumin’s anti-diabetic effects [62]. According to research by Kamar et al., the healing process was enhanced by a curcumin-nanoparticle-loaded hydrogel with complete re-epithelization, an unbroken dermo-epidermal junction, and dermal junction reconfiguration with much more collagen deposition and VEGF and AQP3 production. These findings demonstrated that the application of Cur-NP/HG significantly altered the kinetics of wound healing in diabetic skin wounds compared to those that received Cur/HG [63]. In a Sprague Dawley diabetic rat model, Li et al. (2019) created curcumin-loaded chitosan nanoparticles to improve diabetic wound healing by reducing inflammation. Additionally, they discovered that Cur-CS-NPs might significantly enhance angiogenesis in vivo and in vitro while reducing macrophage-mediated inflammation [64]. Furthermore, Mahmood et al. (2022) developed a curcumin-loaded self-emulsifying drug delivery system and found that it dramatically accelerated wound healing and re-epithelialization compared to the untreated and purely drug-treated groups in an in vivo diabetic wound model [65].

## 5. Curcumin Encapsulated Nano Delivery Systems for Targeting Wound Healing

**Figure 3:**
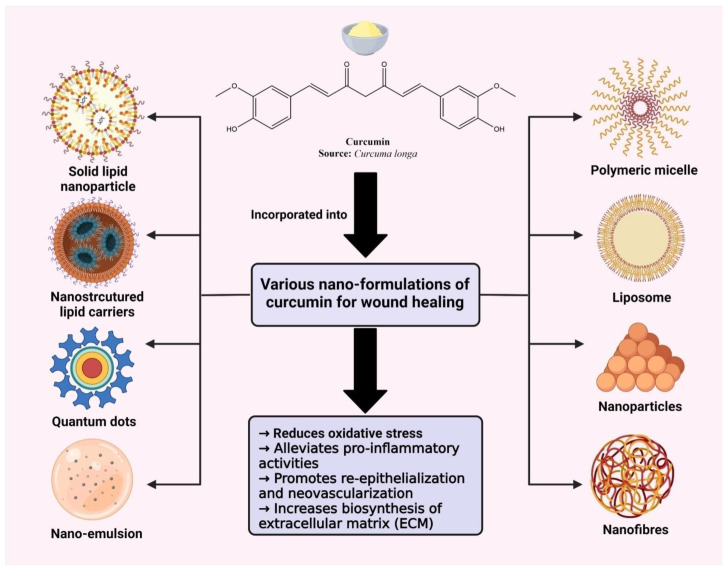
Applications of various topical formulations of curcumin for wound healing.

Curcumin belongs to class IV of the biopharmaceutical classification system, i.e., low solubility and low permeability [66,67]. These properties result in reduced absorption, bioavailability, and stability. Moreover, it shows a very fast metabolism at specific intervals in the body. To overcome these limitations, many formulations have been tried and tested, including nanoparticles, liposomes, nanogels, nano-emulsion, and the use of an adjuvant and nanocrystals (Figure 3) [68,69,70,71]. Nanotechnology has proven to be an effective technique to accelerate wound healing by stimulating appropriate mobility through various healing phases. Nanotechnology-based materials, such as nanomaterials, nano scaffolds, nanofibers, and biomaterials are also popular for the enhanced local delivery of the active constituent [71].

Approximately 500 publications in last two decades have demonstrated the application of curcumin in wound healing. Out of these, nearly 20% of reports were summarized in the last year.

Moreover, researchers have been studying curcumin-loaded nanocarriers (Table 1) to decrease scar formation while maintaining a high rate of wound closure. In the long run, more research is required to understand how research findings about curcumin-based medicines might be utilized in clinical settings.

### 5.1. Liposomes

Liposomes are nano-sized phospholipid bilayered vesicles that load hydrophilic drugs into the inner core and hydrophobic drugs into a liposome’s bilayer. These are easy to prepare, can be loaded with a variety of drugs, are highly biocompatible in nature. Liposomes resemble our biological membranes; liposome bilayers can quickly increase local medication concentrations. Due to the ability of individual phospholipid molecules to penetrate the lipid layers of the stratum corneum (SC) and epidermis, liposomes may function as local depots (reservoir effect) for the prolonged release of dermatological active chemicals [81]. Liposomal compositions with phospholipids may help with drug distribution by enhancing penetration. By fusing or combining with the SC, these substances disrupt the lipid matrix and promote drug flow through the skin. Thereby, liposomes have emerged as an appealing nanocarrier for the delivery of a variety of therapeutics, including wound healing agents [82]. Li et al. investigated a curcumin-based liposomal hydrogel formulated with phospholipase A2 as an enzyme activator for infected wounds [83]. Liposomal hydrogel was prepared by integrating curcumin into the liposomal bilayer and then the formed liposomes were encased in gelatin chitosan hydrogel. The formed formulation prevented drug degradation and improved drug release efficiency to the infected skin area. This developed formulation also showed a controlled release on demand by modulating phospholipase A2 biological activity in accordance with the wound infection. Choudhary et al. utilized a film hydration approach for developing curcumin-loaded liposomes. This approach led to the improved aqueous solubility of curcumin and the development of a sustained and prolonged drug-release system, thus overall adding value to its improved bioavailability [84]. The resulting liposomes had reduced particle size, higher encapsulation efficiency, improved stability, and excellent skin permeation. The developed formulation showed promising results in improvising scar-free and enhanced wound healing and closure. The efficacy of curcumin-based propylene glycol nano liposomes (Cur-PgL) in the treatment of second-degree burns was studied by Kianvash et al. and his colleagues. Without the use of any chemicals that could be potentially lethal, they created Cur-PgL 0.3% and used it to heal wounds in rats. When wounds were treated with Cur-PgL 0.3%, their macroscopic texture was thin, light pink to yellow, and the exudate was completely under control by the seventh day from the day of treatment [85].

### 5.2. Nanoparticles

Nanoparticles (NPs) include those particulate substances which have one dimension at least under 100 nm [86,87,88]. They possess a high surface-to-volume ratio and thus, easily approach the interior cell environment leading to its high therapeutic efficacy. These systems can be modified in size, shape, and chemical properties to ease cellular interactions. Owing to these properties, NPs have become a hot topic for drug delivery. NPs have also become popular for treating infectious disorders, especially skin wounds. Alqahtani M. et al. prepared curcumin-loaded lignin nanoparticles for the fast healing of wounds following tissue injury. The developed NPs significantly increased their wound healing activity in contrast to the curcumin solution and blank NPS in its in vivo investigation. Developed NPs exhibited better wound healing, anti-inflammatory properties, re-epithelialization, and granulation tissue creation with escalated collagen disposition within a 12-day timeframe [89]. Krausz A. E. et al. synthesized and characterized curcumin NPs. The developed NPs showed enhanced wound healing and inhibited the in vitro growth of *Pseudomonas aeruginosa* and methicillin-resistant *Staphylococcus aureus* in a dose-dependent manner in an in vivo murine wound model [90].

### 5.3. Nano-Emulsion

Nano-emulsions (NE) are isotropic mixtures of oil and water stabilized by surfactant or co-surfactant. These are transparent and thermodynamically stable systems. NE are able to permeate the epidermis and subcutaneous barrier of the skin tissue and thus improve solubilization and permeation of the drug incorporated. Ahmad et al. demonstrated curcumin-loaded self-nano emulsifying drug delivery systems to enhance curcumin solubility along with topical wound healing potential [91]. Pathan et al. developed a curcumin-loaded fish scale collagen HPMC nanogel. It was reported with high efficacy and stability in wound healing applications [92]. It was also observed that an ex vivo permeation study revealed that CNG had a prolonged release and had a higher wound contraction value than other formulations and had a non-irritant action. A curcumin-loaded nanoemulsion was developed by Thomas et al. using g Labrafac PG + Triacetin as the oil, Tween 80 as the surfactant, and polyethylene glycol (PEG 400) as the co-surfactant [93]. The created nanoemulsions were assessed for physical and chemical characteristics as well as ex vivo skin penetration and deposition investigations. Additionally, the findings of this study demonstrated the potential of the curcumin-entrapped nanoemulsion gel in wound healing. Furthermore, on the 12th day of the study, the gel was discovered to completely close the wound. In an another study, a curcumin-loaded nanoemulsion was prepared using an ultrasonication technique [94]. It was discovered that in comparison to curcumin dispersed in a conventional hydrogel system, the produced curcumin nanoemulgel demonstrated thixotropic rheological behavior and a significant increase in skin penetrability features. The efficacy study on in vivo wound healing and the histological analysis of samples of healed tissue demonstrates the importance of the nanomedicine-based strategy in enhancing the biopharmaceutical properties of curcumin.

### 5.4. Quantum Dots

The advent of nanotechnology in medicine and medication delivery has facilitated the development of several innovative methods for the precise diagnosis and effective treatment of clinical problems without side effects. With limited emission and a wide spectrum of absorption bands, QDs are colloidal fluorescent semiconductor nanocrystals that range in size from 2 to 10 nm [95]. Currently, research is being conducted on wound healing to increase the bioactivity and broad-spectrum antimicrobial effects of curcumin, which is mostly used in drug delivery. Wu et al. produced quaternized carbon quantum dots (Q-CQDs) using the double-thermal approach from natural curcumin and 2,3-epoxy propyl trimethyl ammonium chloride (GTA), which have a high degree of solubility and stability. According to the experimental results, the Q-CQDs have remarkable broad-spectrum antibacterial activity, which is much higher than that of natural curcumin. Q-CQDs functionalized with -N+(CH_3_)3 exhibited significant adhesion behavior on the bacterial cell membrane, according to research into the antibacterial mechanism of Q-CQDs. The Q-CQDs entrance caused ROS to be produced and the efflux of cytoplasmic DNA and RNA, which caused the bacterial cells to lose their integrity and resulted in the death of the bacteria. Q-CQDs did not exhibit bacterial resistance and did not induce hemolysis or cytotoxicity. In vivo investigations using mouse models and wounds infected with *S. aureus*, *E. coli*, and mixed bacteria showed that Q-CQDs decreased the bacterial population at the wound site, reduced inflammation, and enhanced wound healing. According to these findings, the Q-CQDs may be a viable antibacterial contender for clinical infected-wound-healing applications and even bacteria-resistant illnesses [96].

### 5.5. Lipid Nanoparticles

The last several decades have seen a surge in interest in lipid nanoparticles (LNPs). The two main categories of lipid-based nanoparticles are solid lipid nanoparticles (SLNs) and nanostructured lipid carriers (NLCs). Because they feature benefits, such as a favorable release profile and targeted drug delivery with outstanding physical stability, SLNs were created to solve the shortcomings of existing colloidal carriers, such as emulsions, liposomes, and polymeric nanoparticles. NLCs are modified SLNs that are used in the next generation of lipid nanoparticles to increase stability and capacity loading [97]. These LNPs are made of solid phase lipids and surfactants and range in size from 40 to 1000 nm on average. A surfactant is used as an emulsifier in the dispersed phase. At both body temperature and in the surrounding environment, SLN lipid components are solid. Cutaneous application of lipid nanoparticles offers a number of benefits, including chemical protection of the incorporated materials, enabling the application of labile molecules that are challenging to transport in conventional semi-solid formulations, and improved drug bioavailability due to the potential for controlling the release of molecules and facilitating their penetration and retention of the skin. The latter is explained by the ease with which lipid nanoparticles cling to the SC, enabling encapsulated chemicals to penetrate deeper skin layers. These characteristics are connected to the physiological lipid composition of the SLN and NLC, which can interact with the SC to rearrange its lipids and facilitate molecule penetration. Additionally, the small size of the nanoparticles increases their adhesiveness and surface contact area, facilitating medication absorption through the skin [97,98]. Fabrication of curcumin-loaded solid lipid nanoparticles (CSLNs) for wound healing was designed by Sandhu et al. The Taguchi design, then the central composite design, was used to optimize CSLNs produced by hot, high-pressure homogenization. With a particle size of less than 200 nm, the improved CSLNs demonstrated high assay/drug content (0.6% *w/w*), solubility (6105 times), and EE (75%). The CSLNs had a regulated release, were safe (in vitro and in vivo), photostable, autoclavable, stable for up to one year at 30 °C. Compared to free curcumin dispersion, the CSLNs significantly inhibited the growth of *Staphylococcus aureus* 9144 (MIC: 64 g/mL for planktonic cells; 512 g/mL for biofilm formation; and 2 mg/mL for mature biofilm). This study on curcumin solid lipid nanoparticles was disrupting mature biofilms (CSLNs). While the wound healing potential of CSLNs (integrated into a hydrogel) was studied in vivo, the cell proliferation potential of CSLNs was also assessed in vitro. In both a full-thickness excision wound model and a nitrogen mustard gas model, CSLNs showed noticeably quicker wound closure, better histological and immune-histochemical healing, lower oxidative stress (LPO), inflammation (TNF), and increased angiogenesis (VEGF) as well as antioxidant enzymes, such as catalase and GSH levels. As a result, and notably, for infected wounds, CSLNs present a promising new wound therapy due to their impact on mature biofilm disintegration [99]. Hydrophilic and lipophilic medicines can be enclosed in nanostructured lipid carriers (NLC). In this reported research, an NLC comprising curcumin and epidermal growth factor (EGF) was designed (EGF–Cur-NLC) by Lee et al. A modified water-in-oil-in-water (*w/o/w*) double-emulsion technique was used to produce EGF-Cur-NLC. The EGF-Cur-NLC particles displayed high encapsulation effectiveness (81.1% and 99.4% for EGF and curcumin, respectively) with an average diameter of 331.8 nm. NIH 3T3 fibroblasts and HaCaT keratinocytes were used in two different in vitro cell experiments. The outcomes demonstrated that the NLC formulation of EGF did not lose any of its bioactivity. Additionally, EGF-Cur-NLC enhanced in vitro cell migration, a model for the healing of wounds. Finally, diabetic rats were used to assess EGF-Cur-NLC in a chronic wound model. It was discovered from the outcomes that EGF-Cur-NLC enhanced wound healing and raised antioxidant enzyme activity. Overall, these findings point to the ability of the NLC formulation, which contains curcumin and EGF, to encourage the healing of chronic wounds [100].

### 5.6. Polymeric Micelles

The core/shell structures of polymeric micelles, which have a diameter of 10 to 100 nm, are created by amphiphilic block copolymers. Polymeric micelles are suited for medication delivery due to their intrinsic and adjustable characteristics. Due to several advantageous features, such as their ability to efficiently emulsify a range of poorly soluble pharmaceutical agents, biocompatibility, longevity, high in vitro and in vivo stability, and capacity to accumulate in pathological areas with compromised vasculature [101,102], Zhang fabricated a curcumin-alginate-based nano-micelle (C-A-NM) to lessen the bioavailability issue with curcumin and improve the transfer to the colon area (C-A-NM). The curcumin-release rate based on the gastrointestinal state was assessed following the manufacture of C-A-NM (55.5 nm) and physicochemical analyses using TEM, DLS, and XRD. Additionally, the MTT method was used to assess C-A-NM effects on HCT-8 cell viability at 24 and 48 h, as well as its antibacterial properties. Along with a colonoscopy on the 14th day, the repaired tissue on the 7th and 14th days was evaluated using the Hematoxylin and Eosin method to help explain the effects of wound healing in rats. The protein/collagen concentration andTGF-1/NF-B gene expression were also measured to assess wound healing in the colon. The toxicology findings also showed that C-A-NM at 7.5 mg had no detrimental effects on cell viability. Based on the lowest inhibitory concentration values of 153, 245, and 319 (g/mL), the activity of the bacteria *Staphylococcus aureus*, *Pseudomonas aeruginosa*, and *Escherichia coli* reduced. In contrast to NFB, the usage of C-A-NM promoted TGF1 expression while also increasing protein and collagen in damaged regions. These findings, along with the outcomes of the colonoscopy and histology, revealed that C-A-NM speeds up the healing of wounds. Overall, the findings indicated that the application of C-A-NM, based on collagen production and decreased bacterial activity, can significantly speed up the healing of wounds in the gastrointestinal system [103].

In Bisphenol A-induced diabetic rats, the thin-film hydration approach has been used to develop curcumin-loaded mixed polymeric micelles based on chitosan, alginate, maltodextrin, pluronic F-127, pluronic P123, and tween 80. The outcomes amply supported the created formulation’s capacity to lower high blood glucose levels and lipid profiles (total cholesterol, and triglycerides). When these curcumin-based formulations were used, it maintained the body weight, HDL cholesterol level, other biochemical markers, and sped up wound healing. In comparison to conventional medications and pure curcumin, the newly discovered curcumin-based formulations have demonstrated superior therapeutic potential and outstanding healing performance [104].

## 6. Curcumin-Loaded Polymeric Systems

A steady rise in nano-loaded polymeric systems over the past few years has uncovered new knowledge about wound skin regeneration [105]. These drug carriers promote skin retention, prolong the drug-release time, and protect the medication from deterioration, increasing the therapeutic potential of both biopolymers and synthetic polymers. Films, gels, sponges, membranes made of natural biopolymers, including collagen, chitosan, gelatin, and HA, and synthetic polymers, such as polyester and polyurethane, are utilized as scaffolds for wound treatment [106].

In the context of polymeric systems, hydrogels are the most popular and have been explored for the preparation of wound dressings. Hydrogels hydrate wounds and encourage autolytic debridement. These systems enhance cell migration and absorb wound exudate. Autolytic debridement without harm to epithelial cells is one of the main advantages of these dressings. These are recommended for injuries ranging from dry to mildly exudating and to degrade slough on the wound surface [107,108,109]. Hydrogels mimic the extracellular matrix and enhance oxygen permeability at the wound site, help in expedited healing, and also act as a barrier, thereby minimizing microbial invasion [109]. Bhattacharya D. et al. optimized a hydrogel scaffold of curcumin with a cerium oxide nanoparticle (CNP) for promoting a balanced microenvironment in the injured tissue to accelerate wound healing. The developed hydrogel dressing displayed good loading efficiency and a long-term release of curcumin. A single application of the developed formulation resulted in better wound healing efficacy (78%) and less scarring in seven days when compared to dressings containing only curcumin or CNP. Curcumin, in combination with CNP resulted in increased cell proliferation, higher collagen content, wound maturation, re-epithelialization, and granulation tissue development [110]. In another study, a nanoformulation of curcumin and cerium oxide was incorporated in gelatin-dextran based hydrogel. The developed hydrogel depicted promising antioxidant effects with increased cellular migration. It showed sustained drug release over an extended period at the wound site in the Sprague rat model [111]. Sonamuthu et al. created a hydrogel dressing that contained biocompatible silk protein, curcumin, and L-carnosine. The L-carnosine dipeptide effectively inhibited bacterial infections and decreased the activity of MMP-9 matrix metalloproteinase. The synthetic hydrogel demonstrated anti-inflammatory, ROS-scavenging, and antioxidant action and improved in vivo healing [112]. Katas et al. developed curcumin- and DsiRNA-loaded chitosan nanoparticles incorporated into a Pluronic F-127 thermosensitive gel. The gel formulation showed how effectively the drug release reduced inflammation in diabetic wounds [113].

For the goal of healing wounds, sponges and films have already undergone extensive research. This type of system can significantly promote cell infiltration, migration, and communication due to its large pore size, which ranges from 50 m to millimeters [114]. Films dressings are thin with flexible layers of polymer with or without the incorporation of plasticizer. These are excellent drug delivery systems as they can target sensitive sites, which is impossible with tablets or liquid formulations [10]. Films have been shown to improve drug efficacy and the onset of drug action, along with reducing drug frequency. These are convenient as they are administered through a non-invasive route, are easy to manufacture and transport, and are cost-effective. Due to the availability of a wide array of suitable polymers and advancements in manufacturing technology, films have become popular drug carriers in the pharmaceutical area. Li et al. investigated a curcumin-nanoformulation-loaded methoxy poly(ethylene glycol)-graft-chitosan film for wound healing potential. The film was developed by a solvent/casting evaporation method. It was reported to significantly elevate the re-epithelialization and collagen synthesis at the wound site. The developed composite film was found to possess excellent wound dressing activity and enhanced the topical delivery of curcumin to promote wound healing in a rat model [115]. Duan et al. developed films of curcumin-grafted hyaluronic acid modified pullulan polymers [Cur-HA-SPu] to accelerate wound healing and also help to fight infections. The developed film was found to enhance wound healing and was reported to nearly close the wound after the 21st day of treatment. The cur-HA-SPu polymers were synthesized successfully by an esterification reaction and were characterized by FTIR, NMR, and DSC. It has many advantages, such as a high swelling ratio, quick hemostasis ability, good antimicrobial activity as well as antioxidant properties. Therefore, the Cur-HA-SPu polymer displayed remarkable accelerated wound healing ability and can be applied in the treatment of skin injuries as a potentially effective functional wound dressing material [116]. Curcumin can also be encapsulated with chitosan-based biomaterial to convert into a film delivery system. Formed systems also reported effective antimicrobial properties [117]. Darandale and Vavia used cyclodextrin-based nanosponges to boost the solubility of curcumin; they created the complex of curcumin with a cyclodextrin nanosponge produced with dimethyl carbonate as a cross-linker [118]. In comparison to the free curcumin and cyclodextrin combination, the loaded nanosponges demonstrated a higher solubilization efficiency. The interactions between curcumin and the nanosponges were validated by the characterization of the curcumin–nanosponge complex. Additionally, curcumin’s controlled long-term in vitro drug release and the nonhemolytic nature of the compound were observed.

Fibers are the most used and popular sealing material compared to other delivery systems. This is attributed due to their high area/volume ratio promoting sufficient porosity and liquid evaporation. These systems are also documented as significant in preventing wound contamination. The main advantage associated with these is that their structure can be changed and netted off to allow drug encapsulation and consistent release thereafter. The incorporation of curcumin in fibers has been reported to improve its stability and enhance its wound healing potential. Thailand da Silva et al. developed polycaprolactone and copolymer F-108-based fibers with curcumin using the electrospinning technique. The developed fibers were shown to increase wound contraction by 13% when compared to the control treatment. Developed fibers were found to stimulate re-epithelialization, proliferation, and revascularization for faster wound healing in open excisional wounds performed in a rat model [107]. Nanofibers, because of their flexibility and low weight, have become an ideal advancement in the field of fibers. They are ideal for protecting wounds from any kind of stress or damage [23]. Electrospun nanofibers possess high physico-mechanical strength, extremely small pore size, greater encapsulation efficiency, excellent inter-pore connectivity, high porosity, tunability, high specific surface area, and controlled morphology [119]. Nanofibrous mats imitate the architecture of the extracellular matrix of the skin and, thus, are capable of adhering to cells and facilitating their differentiation, migration, growth, proliferation, and angiogenesis [120]. The nanofibrous membrane is very well admired in tissue engineering [121]. Mahmud M. et al. formulated curcumin nanofibers with polyvinyl alcohol (PVA) by the electrospinning technique. Nanofiber mats showed excellent antibacterial properties against both gram-positive and gram-negative bacteria [122]. By combining polycaprolactone and polyethylene glycol, Mohammadi Z. et al. created chrysin-curcumin nanofibers using the electrospinning technique. Chrysin was added to the solvent in amounts of 5, 10, and 15% (*w/w*) (relative to the ratio of PCL-PEG-PCL) to prepare chrysin-loaded nanofibers. The developed nanofibers showed a significant anti-inflammatory effect at various phases of wound healing. The use of nanofibers in this analysis addressed the issue of the insolubility of chrysin and curcumin-based materials. The point of that study was to introduce chrysin-curcumin-loaded PCL-PEG nano-fibers as an innovative substance to speed up the healing of wounds [123]. Curcumin is also found to act synergistically with silver in managing wound infection and healing when it is converted into an electrospun nanofiber system with chitosan as a polymer [79].

As a result, curcumin-loaded polymeric systems are extensively studied in wound healing because they are effective in promoting rapid wound closure due to a structural arrangement similar to that of typical skin. Additionally, these materials fall into the category of ideal materials due to their biocompatible and biodegradable nature. Further, these polymers are said to have intrinsic wound healing actions through the re-epithelialization of tissue, cell migration, or regeneration [124,125,126]. Thus, these systems loaded with curcumin are expected to accelerate the healing of wounds with a synergistic action [79].

## 7. Scale Up Process and Toxicity Associated with Curcumin

Nanotechnology is a widely explored field in the 21st century. It has piqued public attention because of the demands and applications of nanosystems in many fields of pharmaceutical sciences. Nanosystems, because of their size, shape and structure, possess an array of advantages over the traditional systems. However, at the same time, it suffers from some very sensitive concerns. Exposure of nanosystems in the environment is unavoidable and hence, nanotoxicity investigation is gathering the interest of researchers globally [127,128]. Their unique physicochemical properties enable nanosystems to interact with the biological systems and may lead to unpredictable and undesirable consequences. Hence, a comprehensive investigation into nano-bio interactions must be conducted to ensure the proper and safe use of nano-materials. The majority of curcumin nanoformulations reported in the literature discussed efforts to enhance curcumin delivery in order to address the problems of low solubility, poor absorption, quick metabolism, and restricted bioavailability. These were primarily attained by the use of targeted delivery, controlled release, mucoadhesion, enhanced stability, and greater cellular absorption [129,130]. Therefore, increasing curcumin bioavailability would be accomplished by using nanotechnology for manufacturing curcumin and other nanomaterials into nanoformulations. Although curcumin nano preparation has not been investigated in clinical settings for wound healing, curcumin nano preparation will continue to be a successful method for treating wounds in the future [130,131]. In addition, little is known about the toxicity and biocompatibility of these formulations. To advance the study of curcumin nanoformulations, the gap in this field must be filled. Notably, because of poor encapsulation rates, difficulties in preparation and unique harmful effects on humans, nanoformulations have only been used in preclinical settings (solvents and surfactants). As a result, additional fundamental research and preclinical tests are still required to assess the pharmacological and toxicological effects of nanoformulations containing curcumin. Many curcumin nanoformulations are now still in the laboratory development stages and will likely take some time before they can be produced on a large scale and be used in clinical settings [131]. To advance, investigations need to be made into production technology and clinical instruments, including standardized production, quality control, drug delivery stability, and adverse reaction monitoring to make more progress. Since curcumin nanoparticles are not tissue-specific, greater thought can be given to developing nano-drug-based tissue-specific delivery systems. Unwanted side effects of nanomedicine-based drug delivery systems include allergic responses, DNA damage, excitotoxicity, and neuroinflammation. Therefore, a detailed investigation and documentation of the biodegradability and biocompatibility of curcumin nanodrugs is required. Additional preclinical and clinical studies are required to fully comprehend the mechanism of action of curcumin nanoformulations. Enhancing curcumin’s antibacterial and anti-inflammatory properties, as well as its nanoformulation, which will deliver its wound healing action, is another viable solution [129,130,131,132]. Then, pharmacological candidates for the treatment of chronic wounds could be developed using the available knowledge. A significant problem is creating a pharmaceutical-grade curcumin nanoformulation and scaling it up successfully. It might be appropriate for a top-down strategy that uses the milling method to nanosize bulk curcumin (a traditional process used to produce drug nanocrystals). The already-established field of technology known as NanoCrystals^®^ can be used to create curcumin nanoformulations [133,134]. It is possible that this approach will not provide much monodispersity of nanoparticles (which often leads to polydispersity). In light of all these factors, a possible alternative strategy is a bottom-up approach-based nanoparticle production with curcumin encapsulation. Curcumin nanoformulations are created by a number of businesses, including, Konark Herbals & Health Care (Mumbai, India), Advanced Orthomolecular Research (Calgary, AB, Canada), Lee Silsby Compounding Pharmacy (Beachwood, OH, USA), Sabinsa Corporation (Piscataway, NJ, USA), etc. Particularly active in curcumin C3 complexes research and development is Sabinsa Corporation. Before being used, their nanoformulations were additionally tested for endotoxin analysis, particle size and zeta potential, in vitro cellular cytotoxicity, immunological assays, metabolism, and pharmacokinetic investigations in cooperation with the Nanotechnology Characterization Laboratory [133,134,135]. These are important biological processes and physicochemical characteristics to take into account while creating an effective curcumin nanoformulation for therapeutics.

Regulatory aspects: The FDA continues to evaluate nanotechnology products on a case-by-case basis, using the combination product framework to identify the type of product and subsequent regulatory requirements. As nanomedicine develops, the FDA faces several difficulties, but three primary difficulties stand out. The first is the suitability of the legal framework itself; nanomedicine emphasizes the rigor of the product domains that set the standards for product approval. Secondly, there is the possibility of unexpected dangers, which calls into question the validity of conventional safety and efficacy requirements and lastly, whether or not consumers can learn whether a product contains nanotechnology or nanomaterials from the labelling of nanomedicine products. FDA must consider whether greater patient and consumer education and engagement are necessary, as well as whether the FDA policy on labelling requirements for nanoproducts adequately addresses public opinion and the needs of the public in terms of health literacy. Large-scale research projects are being conducted to describe nanoscale materials and quantify their impact with the goal of establishing toxicological evaluation and testing tools as part of the National Nanotechnology Initiative and other federal agency collaborations [136]. Additionally, the US Food and Drug Administration (FDA) acknowledges the significance of introducing superior, cutting-edge wound care products to the market. Recent discussions by the FDA included three subcategories of wound dressings that contain medicines, solid wound dressings (such as bandages and gauzes, gels, creams, and ointments), and liquid wound washes, and explicitly addressed the clinical usage and regulatory implications for these products. At present, the 510(k) process for clearing wound dressings containing pharmaceuticals has been predicated on the idea that the drug component is meant to stop the growth of microbes on the device rather than to directly affect the patient [137].

## 8. Patent Technology

The number of patients detected with chronic wounds is rising globally, pressuring the ubiquitous development of wound healing innovations developed for various wound settings. Patent data is the primary source of knowledge; results extracted from patents are crucial in determining modern technological patterns. (Table 2 gives a description of patents). Patent interpretation is frequently used to assess the competitive market in technical variations at an advanced manufacturing or national level, to determine positive technological aspects, and to examine international market potentiality. Furthermore, patent analysis makes it simple to predict future advancements of a specific industry. Because of the large number of people being diagnosed with diabetes as well as obesity, the demand for the rapid healing process has increased substantially. Besides that, innumerable wound-healing innovations are being enhanced to manage this high demand [138].

In patent WO2014147638A1, the inventors claimed that curcumin particles and tulsi extracts are utilized in the formulation matrix to further increase the qualities of the herbal medicinal values and to give a synergistic effect, whereby all the ingredients cooperate to produce better healing outcomes [139]. Similarly, the invention is described in US20190060253A1 which represents a formulation of curcuminoid and turmeric essential oil. Furthermore, this patent describes that this formulation increases the bioavailability of curcumin and its biological activity [140]. It describes how to apply curcuminoid with turmeric essential oil to increase the bioavailability of curcumin for oral supplementation against various disorders and for the management of wound healing. Moreover, the use of turmeric to speed up the healing of wounds is the focus of the current invention, USOOS40504A [141]. It is hypothesized that turmeric works to cure chronic ulcers by enhancing microcirculation, promoting angiogenesis, encouraging the creation of granulation tissue, and speeding up the process of re-epithelialization. Any kind of injury to the body that causes the skin or other tissue to be broken, cut, pierced, or ripped, etc., can be treated, including surgical wounds (such as incisions), ulcers, and other types of external injuries. This patent also describes a technique for encouraging the healing of a wound in a patient that primarily involves giving the patient a wound-healing compound made of a sufficient quantity of turmeric powder. US20050181036A1 describes the development of an aerosol containing lipids and curcumin particles [142]. In this, curcumin is disseminated in a lipid vehicle and an aqueous solvent in the pharmaceutical compositions of the present invention, making them suitable for aerosol delivery to a subject. Small aqueous aerosol particles with lipids, lipid complexes, liposomes, and interacting curcumin-lipid or curcumin-liposomes are the subject of the present invention. Various combinations of particles are propelled or transported by air or air with added oxygen. It is noted that curcumin, one or more lipids, and an aqueous solvent can be combined to create pharmaceutical lipid vehicle compositions of curcumin that are suitable for aerosol delivery to a subject, provided that the transition temperature of the lipid if only one lipid is present, or the mean transition temperature of the lipids, if more than one lipid is incorporated, is less than about 15 °C and the composition can be nebulized.The methods of the present invention can be used to speed up the healing of wounds and stop wrinkle formation on the skin. This data significantly shows that curcumin-based formulations have a hope for market commercialization.

**Table 2:** Description of numerous patents on curcumin-based formulations for wound healing.

Patent No.	Title	Date of Publication	Description	Assignee	Reference
US20210322458A1	Methods and compositions for treatment of burns, joint pain, and fungal infections	21 October 2021	Disclosure relates to various pharmaceutical compositions, some of which incorporate curcumin for the treatment of burns, e.g., sunburns, methods of making same, and methods of treating burns using same.	Gregg Tobin, Greg Glaze	[143]
WO2021140366A1	Means to improve usability of a wound insert for application to deep wounds	15 July 2021	The present disclosure relates generally to wound inserts that may include an outer layer and an inner core of biopolymers that may be used in the therapy of tunneling wounds and for facilitating wound healing. Kits for use in practicing the methods are also provided.	Kci Licensing, Inc.	[144]
EP3943096A1	Method of synthesizing a turmeric-based composition having enhanced bioavailability	26 January 2022	The present invention relates to a method of synthesizing a turmeric-based composition wherein a method to enhance bioavailability of curcumin is discussed.	Star Hi Herbs Pvt Ltd.	[145]
WO2021173933A1	Formulations and uses thereof	2 September 2021	It includes preparation and formulation containing curcumin capable of crossing and incorporating into a membrane of a cell or an organelle or an exosome are described.	Ma Joyce H	[146]
WO2021176327A1	Liposomal compositions of curcumin and process for preparation thereof	10 September 2021	The present invention relates to a liposomal composition comprising curcumin as an active ingredient for the treatment of various inflammatory disorders.	Yogesh Found	[147]
WO2021225548A1	A novel method for carrying bioactive molecules using nanocarriers	11 November 2021	In the present invention, many bioactive molecules, especially curcumin, can be carried to target tissues in the living organism by using a nanocarrier. It can be used in all sectors for carrying molecular structures at nano size.	OzmenZekeriya	[148]
WO2021225918A1	Semi-solid chewable compositions and methods of making and using thereof	11 November 2021	A semi-solid chewable composition, comprising an herbal composition and a semi-chewable base. The herbal composition comprises anti-inflammatory, anti-microbial, analgesic, antiseptic, or antipyretic herbal composition.	Seattle Gummy Company	[149]

## 9. Conclusions and Future Perspectives

Curcumin is a potent anti-inflammatory and antioxidant compound. Despite the aforementioned benefits, it has limitations, including less stability and poor bioavailability. Combinational therapy and the nanoformulations have been used to confront these respective limitations. This review encompasses the investigational indication to provide insight into the wound healing potential of curcumin. For many years, curcumin topical formulations and nano-architectures have been designed and evaluated to enhance curcumin’s wound-healing ability. The primary reason for favoring the topical nanoformulations of curcumin is that it provides optimal solubility, improved bioavailability, and controlled release of curcumin in an active component, which is probably helpful for supplying a continuous dose of medication for extended periods to enhance the healing of wounds. The molecular processes that control the genetic and cellular wound environment and restore chronic inflammation are yet unclear. Therefore, additional study is required to translate the knowledge gained from using animal models into people to develop innovative, efficient, and secure nanodrugs for the treatment of wounds. Recognizing the optimum dosage of curcumin is highly crucial for various targets, most notably its intricate involvement in the inflammatory process and in tissue regeneration, which must be acknowledged before any further drug trials. Even though conventional research on various topical formulations of curcumin appears convincing, the majority of study reports are gleaned from in vitro and in vivo studies, but clinical trials are still required. In recent years, clinical methods have evolved to include trial designs, patient populations, accurate wound assessments, and sophisticated instruments. Findings of the clinical trials may encourage professionals to conduct further research into developing curcumin-based formulations. Additionally, developing more affordable, scalable nanoencapsulation techniques for curcumin is an industrial imperative for lowering production costs and improving competition with other drugs. Hence, nanoformulations of curcumin may prove valuable in the near future for various bioproducts, but more research is required, particularly on wound healing, to provide scientists with a deeper understanding. Overall, additional studies are required to determine the clinical efficiency of curcumin-based formulations to move the technology from the laboratory to the bedside.

## Figures and Tables

**Table 1:** Recent research studies on curcumin-loaded nanoformulations for the treatment of wounds.

Components Incorporated	Formulation Developed	Characterization Techniques	In Vivo Model	Inference	Reference
Curcumin, carboxymethyl guar gum and reduced graphene oxide	Nanocomposites	FTIR, TGA, XRD	Rabbits	The proliferation of fibroblast cell lines 3T3-L1 resulted in 100 percent wound closure and regulated drug release. In addition, in vivo experiments revealed that the CMGG, rGO nanocomposite with curcumin had a wound-healing ability along with antibacterial, anti-inflammatory, and antioxidant effects.	[72]
Curcumin, chitosan, gelatin, PCL	Nanofibrous membrane	SEM, FTIR	Rats	It caused granulation tissue growth, collagen deposition, and epithelial tissue remodeling. It could also speed wound healing by facilitating the expression of CD31 and TGF- in the early stages of the wound, and improved antioxidant properties.	[73]
Curcumin loaded PCL/P VA-silk fibroin	Nanofibrous mat	SEM	Albino mice	Showed excellent antioxidant and anti-inflammatory action, along with accelerated wound-healing.	[74]
Curcumin, resveratrol, tween 80, and labrafac PG	Nanoemulsion	Particle size, PDI, zeta potential, reversed-phase HPLC analysis	Adult male Wister rats	In the skin of burnt rats, the developed formulation led to an increase in antioxidant and anti-inflammatory potential, as well as an increase in collagen and amino acid levels.	[75]
Zinc-Aluminium-LDH-Curcumin	Nanocomposite	TGA, DTA, XRD, FESEM, HRTEM, EDX	Adult male albino rats	Showed anti-inflammatory properties of both LDH and curcumin, as well as their biocompatibility with living matter, expanding their biomedical applications in this era with safety and efficacy through sustained drug release.	[52]
Curcumin, gelatin, sodium bicarbonate and honey	Nanofibrous membrane	SEM, FTIR	Wistar male albino rats	Accelerated the wound healing process by promoting re-epithelialization, proliferation of fibroblasts and providing anti-inflammatory action.	[76]
Surfactin, PCL gel and curcumin	Nanocomposite	FTIR, SEM, Wettability	Male Wistar rats	Increased curcumin’s bioavailability as an anti-inflammatory agent and accelerated different stages of wound healing. Furthermore, the produced dressings showed good bio-compatibility.	[77]
Heparin-PLGA Curcumin, EDC, and NHS	Nanofiber membrane	XPS analysis, water contact angle, WVTR, FE-SEM	Sprague Dawley (SD) rats	Increased hydrophilicity, resulting in faster cell migration and antioxidant activity.	[78]
Curucmin-loaded β-cyclodextrin, AgNPs chitosan, and polyethylene oxide	Nanofibers	Zeta potential, SEM, FTIR, AFM	Male Kunming mice	Stimulated skin wound healing by controlling angiogenesis and increasing proliferation of surrounding tissue, as well as reducing scar tissue formation.	[79]
CUR loaded CH/PEG/Ag	Nanoparticles	UV-Vis spectroscopy, XRD, FTIR, FESEM, TEM, TGA	Wistar albino rats	Developed formulation showed complete tissue regeneration, as well as the prevention of microbial infections in wounds, the quick healing of wounds, and the inhibition of apoptotic cell growth was observed.	[80]

## Abbreviations

AFM	Atomic Force Microscopy
BBB	Blood Brain Barrier
DTA	Differential Thermal Analysis
EDC	1-(3-dimethylaminopropyl)-3-ethylcarbodiimide
EDX	Energy dispersive X-ray
FESEM	Field Emission Scanning Electron Microscopy
FTIR	Fourier Transform Infrared Spectroscopy
HPLC	High-Performance Liquid Chromatography
NHS	N-hydroxysuccinimide
NFκB	Nuclear Factor kappa B
NLC	Nanostructured Lipid Carriers
Nrf2	Nuclear factor erythroid 2-related factor
PDI	Polydispersity index
ROS	Reactive Oxygen Species
SEM	Scanning Electron Microscopy
SLN	Solid Lipid Nanoparticle
STAT	Signal Transducer and Activator of Transcription
TEM	Transmission Electron Microscopy
TGA	Thermogravimetric Analysis
WVTR	Water Vapor Transmission Rate
XRD	X-ray powder diffraction (XRD)
